# Comparison of Online Patient Reviews and National Pharmacovigilance Data for Tramadol-Related Adverse Events: Comparative Observational Study

**DOI:** 10.2196/33311

**Published:** 2022-01-04

**Authors:** Susan Park, So Hyun Choi, Yun-Kyoung Song, Jin-Won Kwon

**Affiliations:** 1 BK21 FOUR Community-Based Intelligent Novel Drug Discovery Education Unit, College of Pharmacy, Research Institute of Pharmaceutical Sciences Kyungpook National University Daegu Republic of Korea; 2 Department of Statistics Kyungpook National University Daegu Republic of Korea; 3 College of Pharmacy Daegu Catholic University Gyeongsan-si, Gyeongbuk Republic of Korea

**Keywords:** drug safety, pharmacovigilance, tramadol, social media, adverse effect

## Abstract

**Background:**

Tramadol is known to cause fewer adverse events (AEs) than other opioids. However, recent research has raised concerns about various safety issues.

**Objective:**

We aimed to explore these new AEs related to tramadol using social media and conventional pharmacovigilance data.

**Methods:**

This study used 2 data sets, 1 from patients’ drug reviews on WebMD (January 2007 to January 2021) and 1 from the US Food and Drug Administration (FDA) Adverse Event Reporting System (FAERS; January 2016 to December 2020). We analyzed 2062 and 29,350 patient reports from WebMD and FAERS, respectively. Patient posts on WebMD were manually assigned the preferred terms of the Medical Dictionary for Regulatory Activities. To analyze AEs from FAERS, a disproportionality analysis was performed with 3 measures: proportional reporting ratio, reporting odds ratio, and information component.

**Results:**

From the 869 AEs reported, we identified 125 new signals related to tramadol use not listed on the drug label that satisﬁed all 3 signal detection criteria. In addition, 20 serious AEs were selected from new signals. Among new serious AEs, vascular disorders had the largest signal detection criteria value. Based on the disproportionality analysis and patients’ symptom descriptions, tramadol-induced pain might also be an unexpected AE.

**Conclusions:**

This study detected several novel signals related to tramadol use, suggesting newly identified possible AEs. Additionally, this study indicates that unexpected AEs can be detected using social media analysis alongside traditional pharmacovigilance data.

## Introduction

Tramadol is a synthetic analgesic. It is a weak μ-opioid receptor agonist and is considered a different class of analgesic to conventional opioids [1,2]. Tramadol has earned a reputation for fewer side effects and lower rates of respiratory depression, overdose, and addiction due to its lower affinity with μ-opioid receptors than other opioids [3,4]. As a result, tramadol prescriptions have rapidly increased over the years [5-7] and it is extensively prescribed for many types of pain [6-8]. However, there is insufficient empirical evidence that tramadol is safer than other opioids. Recent systematic reviews have revealed that tramadol is more likely to induce severe adverse events (AEs) such as seizures and hypoglycemia than other opioids [9-11]. Some studies suggest that tramadol has a similar or higher risk of long-term opioid use than other short-acting opioids [12,13]. Furthermore, a recent study found tramadol to be associated with increased mortality risk [14,15].

The traditional pharmacovigilance method of data acquisition from spontaneous reporting systems is often used to detect AEs [16]. However, this method is limited by under-reporting as it is known that fewer than 10% of AEs are reported [17]. Recently, internet-based AE detection has been used as a data-gathering tool complementary to traditional pharmacovigilance. Patients share their treatment experiences online, including drug side effects. Several studies have utilized online patient reviews for the purposes of pharmacovigilance [18,19].

In light of increasing evidence of risks associated with tramadol and the limitations of spontaneous reporting systems, this study aimed to explore new evidence for tramadol-related AEs using online patient reviews alongside national pharmacovigilance data. Additionally, we assessed the usefulness of online patient reviews in monitoring tramadol-induced AEs.

## Methods

### Data

The study used 2 data sources to collect information about tramadol-related AEs: (1) the US Food and Drug Administration (FDA) Adverse Event Reporting System (FAERS); and (2) posts in a health forum, WebMD. First, we used FAERS data as traditional pharmacovigilance data to find new AE signals. Globally, the United States is the country with the highest number of opioid analgesics consumed [20], and FAERS is the representative spontaneous reporting system with the largest number of publications related to tramadol-related AEs [21,22]. FAERS contains information about AEs and medication error reports submitted to the FDA, coded in the Medical Dictionary for Regulatory Activities (MedDRA) terminology. Reporting to FAERS is voluntary for health care professionals and consumers, while it is mandatory for manufacturers [23]. Next, WebMD, one of the popular health forums, was chosen to obtain more detailed patient narratives of AE symptoms. The AEs were investigated using various types of social media such as social networking sites (Facebook, Twitter), blogs, forums, and comments [24]. Among them, the health forums have the advantage of effectively obtaining patient experiences online because they are specialized medical topics and have many patient users [25]. WebMD is a well-known health information service website in the United States that provides information on health and wellness topics, including drug information [26]. It also provides bulletin boards for posting personal reviews on specific drugs, frequently used as data sources for AE-related research [24,25].

### Demographics

We used personal information on age and sex for FAERS and WebMD data sets; additionally, occurrence country was used for FAERS data in the analysis. FAERS provides various personal information, including age, sex, occupation, and country where the AEs occurred in the demographic file. All data sets were released after anonymization according to the privacy policy of the US Department of Health & Human Services [27]. WebMD displays the patient reviews of specific medications with nicknames, ages, and sex. WebMD also has its privacy policy fully complying with data protection regulations and developed to provide a safe space for sharing health-related information online [28]. We carefully considered data security and user privacy, and this study was ethically approved by the KNU Institutional Review Board (KNU-2021-0401)

### FAERS Adverse Event Reports

We downloaded AE reports for January 2016 to December 2020 from the FAERS website [29]. From the 7,843,727 reports, we identified duplicate reports by case ID and selected the most recent to eliminate duplicate data. This left us with 6,874,999 reports. From these, tramadol-related AEs were retrieved for both single- and multi-ingredient drugs, including searches of the various brand names used in different countries as well as the generic drug name (n=92,135). Finally, we excluded AE reports in which tramadol was not reported as the primary or secondary suspected drug to rule out the potential misclassification of other drug-related AEs (n=29,345). The specific drug names and numbers of AE reports by year are summarized in Multimedia Appendix 1.

### WebMD Adverse Event Reports

Online patient reviews of tramadol from September 2007 to August 2020 were gathered from the WebMD website using a Python web crawler. Patient review data were collected for tramadol HCL as well as combination drugs using both generic and brand names. A total of 3917 posts were automatically collected. Most of these pertained to tramadol HCL (n=2762). For the combination drugs, the majority of posts pertained to Ultram (Ultram, n=704; Ultram ER, n=134; Ultracet, n=177; tramadol HCL-acetaminophen, n=132; Conzip, n=8). Some of the posts only provided a rating of the drug. These were excluded and we retained only those posts with detailed descriptions. We then excluded posts that did not relate to drug-related AEs, such as drug prescription information and advertisements. Finally, we reviewed the remaining 2062 posts and assigned annotations according to the types of AEs described. The collected data were independently reviewed by 2 researchers (SP & J-WK), and symptoms considered as AE were manually assigned preferred terms (PTs) for AE types from MedDRA. If the PTs assigned by the 2 researchers did not match, a PT was agreed upon through discussion.

### Statistical Analysis

To compare the distribution of tramadol-related AEs reported on FAERS with that of those reported on WebMD, we first categorized reports into respondent age groups. The age groups were children and adolescents (ages <19 years), adults (ages 19-64 years), and older adults (ages ≥65 years). The number of AEs was counted based on MedDRA’s PTs in both data sets. Two or more PTs reported in a patient were counted as different AEs. The distribution of AEs was explored using the system organ classes (SOCs) of the MedDRA.

Additionally, we performed a disproportionality analysis of the FAERS data to identify the detection of tramadol-related AEs in traditional pharmacovigilance data. Disproportionality analysis is a comparison of observed and expected values; in this case, for tramadol-related AEs [30]. We calculated the proportional reporting ratio (PRR), the reporting odds ratio (ROR), and the information component (IC), which are commonly used as disproportionality measures. The threshold criteria for signal detection of adverse drug reactions were defined as PRR ≥2, ROR ≥2, and the observed number of tramadol-related AEs ≥3. The signal criterion for the IC measurements was when the lower limit of the 95% CI was greater than 0.

New signals were defined as AEs satisfying the signal criteria that were previously unknown or incompletely described. Their status as new was ascertained from tramadol label information. The drug labels were retrieved from a regulatory authority database (FDALabel, FDA, USA) and medical resources software systems (UpToDate, Wolters Kluwer Health; Micromedex, IBM). Medical events such as death, disability, hospitalization, or life-threatening consequences were classed as serious AEs. All statistical analyses were performed using SAS, version 9.4 (SAS Institute Inc.).

## Results

Table 1 shows the age and gender distributions of the patients with tramadol-related AEs from the WebMD and FAERS data sets. The majority of those who reported AEs on WebMD were adults aged 19-64 years. There were more reports from women than men. The FAERS data set showed a higher proportion of AEs reported by male and elderly patients. FAERS data had more missing values than WebMD data. The number of patient reports on FAERS was 10 times higher than on WebMD (n=2062 on WebMD; n=29,345 on FAERS). The majority of AEs in FAERS were reported from North America and Europe. The country distribution could not be presented on WebMD due to the lack of information.

Because some patients had multiple tramadol-related medical events, the number of AEs was greater than the number of patients. A total of 4288 and 123,393 AEs were reported on WebMD and FAERS, respectively. This corresponds to an average of 2.1 and 4.0 PTs per patient on the WebMD and FAERS systems, respectively. Table 2 shows the SOC of tramadol-related AEs for the 2 data sets. The SOC with a large number of AEs reported was largely consistent between the 2 data sets, although the frequency rankings were slightly different. There were many reports of psychiatric disorders, general disorders/administration site condition, nervous system disorders, and gastrointestinal disorders. However, the FAERS data included a higher percentage of injury, poisoning, and cardiac disorders. WebMD reports included a higher percentage of metabolism/nutritional disorders and eye disorders. FAERS and WebMD reported 22 and 27 SOCs, respectively, from a total of 27 possible MedDRA SOCs (data not shown).

Table 3 presents the distributions of AEs by MedDRA PTs of the WebMD and FAERS data sets in more detail. Drug inefficacy and drug dependence were the most frequently reported AEs on WebMD and FAERS, respectively. Patients from both data sets commonly reported drug dependence, nausea, vomiting, insomnia, dizziness, fatigue, anxiety, seizure, and pain. FAERS had a higher proportion of overdose, toxicity with various agents, death, and completed suicides. By contrast, WebMD reports included higher percentages of pruritus, constipation, and hyperhidrosis. Most reported AEs from both sources corresponded to those described on the drug labels. However, tramadol-related pain was not on these labels.

**Table 1:** Demographic characteristics of patients from the WebMD and FAERS^a^ data sets.

Characteristics		WebMD, n (%)	FAERS, n (%)
Number of patients		2062 (100.0)	29,345 (100.0)
**Age**			
	Less than 19 years old	12 (0.58)	1120 (3.82)
	19-64 years old	1749 (84.82)	10,539 (35.91)
	More than 64 years old	239 (11.59)	5523 (18.82)
	Unknown	62 (3.01)	12,163 (41.45)
**Gender**			
	Women	1359 (65.91)	14,928 (50.87)
	Men	598 (29.00)	10,704 (36.48)
	Unknown	105 (5.09)	3713 (12.65)
**Occurrence country**			
	Africa	N/A^b^	96 (0.33)
	Asia	N/A	1298 (4.42)
	Europe	N/A	12,426 (42.34)
	North America	N/A	14,997 (51.11)
	Oceania	N/A	289 (0.98)
	South America	N/A	141 (0.48)
	Unknown	N/A	98 (0.33)

^a^FAERS: US Food and Drug Administration (FDA) Adverse Event Reporting System.

^b^N/A: not applicable.

**Table 2:** Classification of frequently reported tramadol-related adverse events from the WebMD and FAERS^a^ data sets.

WebMD (N=4288)			FAERS (N=123,393)		
SOC^b^	Value, n (%)	SOC		Value, n (%)	
Number of AEs	4288 (100.0)	Number of AEs		123,393 (100.0)	
General disorders and administration site conditions	1360 (31.72)	Psychiatric disorders		23,340 (18.92)	
Psychiatric disorders	900 (20.99)	General disorders and administration site conditions		16,899 (13.70)	
Nervous system disorders	827 (19.29)	Injury, poisoning and procedural complications		15,764 (12.78)	
Gastrointestinal disorders	568 (13.25)	Nervous system disorders		14,826 (12.02)	
Skin and subcutaneous tissue disorders	239 (5.57)	Gastrointestinal disorders		9315 (7.55)	
Investigations	80 (1.87)	Respiratory, thoracic and mediastinal disorders		4839 (3.92)	
Musculoskeletal and connective tissue disorders	64 (1.49)	Musculoskeletal and connective tissue disorders		4729 (3.83)	
Respiratory, thoracic, and mediastinal disorders	48 (1.12)	Investigations		4068 (3.30)	
Metabolism and nutrition disorders	40 (0.93)	Skin and subcutaneous tissue disorders		3665 (2.97)	
Eye disorders	32 (0.75)	Cardiac disorders		3274 (2.65)	
Others	130 (3.03)	Others		22,674 (18.38)	

^a^FAERS: US Food and Drug Administration (FDA) Adverse Event Reporting System.

^b^SOC: system organ classes of the Medical Dictionary for Regulatory Activities.

**Table 3:** Frequently reported tramadol-related adverse events from the WebMD and FAERS^a^ data sets.

WebMD (N=4288)			FAERS (N=123,393)	
PT^b^	Value, n (%)	PT		Value, n (%)
Drug ineffective	599 (13.97)	Drug dependence		5547 (4.50)
Withdrawal syndrome	263 (6.13)	Overdose		3506 (2.84)
Insomnia	219 (5.11)	Toxicity to various agents		2824 (2.29)
Nausea	218 (5.08)	Drug hypersensitivity		2162 (1.75)
Drug dependence	197 (4.59)	Drug abuse		2053 (1.66)
Dizziness	197 (4.59)	Death		1840 (1.49)
Headache	171 (3.99)	Pain		1703 (1.38)
Somnolence	141 (3.29)	Nausea		1689 (1.37)
Pruritus	121 (2.82)	Drug ineffective		1673 (1.36)
Drug tolerance	105 (2.45)	Vomiting		1549 (1.26)
Vomiting	101 (2.36)	Completed suicide		1292 (1.05)
Abdominal pain (upper)	83 (1.94)	Somnolence		1247 (1.01)
Constipation	81 (1.89)	Dizziness		1046 (0.85)
Seizure	73 (1.70)	Depression		957 (0.78)
Fatigue	71 (1.66)	Confused state		954 (0.77)
Hyperhidrosis	68 (1.59)	Fall		920 (0.75)
Tremor	66 (1.54)	Anxiety		893 (0.72)
Feeling abnormal	64 (1.49)	Seizure		874 (0.71)
Pain	57 (1.33)	Fatigue		839 (0.68)
Anxiety	52 (1.21)	Headache		838 (0.68)
Euphoric mood	52 (1.21)	—		—
Others	1289 (30.06)	Others		88,987 (72.12)

^a^FAERS: US Food and Drug Administration (FDA) Adverse Event Reporting System.

^b^PT: preferred term of the Medical Dictionary for Regulatory Activities.

In Table 4, we present some examples of patient reports of tramadol-related pain from WebMD. Tramadol-related pain was of 2 types: pain caused by drug use and pain caused by drug discontinuation. The first 3 cases shown in the table are type 1, in which patients complained of pain after taking tramadol. Type 1 pain was primarily reported by patients who had been using tramadol for less than 1 year. The next 3 cases on the table are type 2, in which pain appears to be a symptom of withdrawal resulting from drug discontinuation, mostly in long-term tramadol users.

**Table 4:** Examples of patient reports of tramadol-related pain from WebMD.

Type	Gender	Age	Treatment duration	Medicine	Extracts text from review posts^a^
1	Female	45-54	1-6 months	Tramadol HCL	My doctor recommended this medication to me because of a pain I was experiencing. I went through a severe experience of drowsiness and more pain that I was even fainting. I had never used it again.
1	Female	65-74	Less than 1 month	Tramadol HCL	It helped for a couple days than my mouth & tongue broke out in sores and I started get upset stomach & worsen pain
1	Female	35-44	1-6 months	Ultram	I have cervical dystonia they gave me this pill that did nothing for pain. The pain got worse and worse finally on something different...
2	Female	25-34	1-6 months	Ultram	Wasn’t for me. I can see how this could work for some but not for me. It took care of knee pain the first 2 days. The first day it even fixed a headache. After the third day, it did take the edge off but I still had stiffness and bruising type feeling on the knee. When off of the medicine, the pain was severe and sharp. Overall, it did work to help through the everyday but 3 flights up and down a few times a day isn’t any match for this medicine.
2	Female	19-24	2 to less than 5 years	Tramadol HCL	I have been taking tramadol for the past 3 years and I agree that it does help with the pain. But after about the first year it stops working as well and you want to take more of the drug. It is very habit forming. There are too many withdrawal effects to mention. Sweating-freezing.. Diaharrea vomiting..sleepless nights.. Pain in the legs.. Word of advice don’t take this if you haven’t already! You dont want the side effects! You will be sorry!
2	Female	45-54	10 years or more	Ultram	I can’t stop taking it. The pain has got worse with this drug. The withdrawal is terrible. Side effects are flushing, sweating, irritable, over sensitive, pain, suicidal, can’t spell (hehe)

^a^The reports are presented without correction in their original form as posted on the website.

In the FAERS data set, a total of 860 tramadol-related AEs satisﬁed all 3 of the signal detection criteria: PRR, ROR, and the lower limit of IC. Among them, 125 new signals not listed on the drug labels were found. The tramadol-related AEs and the PRR, ROR, and IC values are presented in Multimedia Appendix 2. The 3 tramadol-related AEs with the highest signal detection criteria values were ligament calcification (PRR=348.9; ROR=349.0; IC=4.1), hematidrosis (PRR=232.6; ROR=232.6; IC=3.1), and neurovascular conflict (PRR=139.6; ROR=139.6; IC=2.7). We also detected 20 new serious AE signals from the following 6 SOC categories: respiratory, vascular, gastrointestinal, infections, musculoskeletal, and nervous system disorders (Table 5). The vascular disorders category showed the largest criteria value of signal detection of the new serious AEs. Femoral artery aneurysm and iliac artery occlusion were included as serious AEs in this SOC category. Our results also included incidents of serious cardiotoxicity, intestinal twists or perforation, infections of the heart or lungs, and paralysis.

**Table 5:** New types of tramadol-related serious adverse events from the FAERS^a^ data set.

SOC^b^ and PT^c^			PRR^d^		ROR^e^		IC^f^
**Respiratory, thoracic, and mediastinal disorders**							
	Diffuse alveolar damage	15.0		15.0		3.5	
**Cardiac disorders**							
	Toxic cardiomyopathy	22.9		22.9		3.0	
	Cardiorenal syndrome	10.0		10.0		2.8	
	Kounis syndrome	5.4		5.4		2.2	
**Vascular disorders**							
	Femoral artery aneurysm	74.8		74.8		3.9	
	Iliac artery occlusion	34.5		34.5		3.8	
**Gastrointestinal disorders**							
	Splenic artery aneurysm	15.8		15.8		2.5	
	Volvulus	4.0		4.0		1.8	
	Large intestine perforation	2.5		2.5		1.2	
**Infections and infestations**							
	Cardiac infection	5.5		5.5		2.0	
	Empyema	4.6		4.6		2.0	
	Endocarditis	3.5		3.5		1.7	
**Musculoskeletal and connective tissue disorders**							
	Rhabdomyolysis	3.7		3.7		1.9	
**Nervous system disorders**							
	Cerebellar infarction	4.9		4.9		2.0	
	Toxic encephalopathy	3.2		3.2		1.6	
	Quadriplegia	4.5		4.5		2.0	
	Hemiplegia	2.7		2.7		1.4	
	Paraplegia	2.3		2.3		1.1	
	Paralysis	2.1		2.1		1.0	

^a^FAERS: US Food and Drug Administration (FDA) Adverse Event Reporting System.

^b^SOC: system organ classes of the Medical Dictionary for Regulatory Activities.

^c^PT: preferred term of the Medical Dictionary for Regulatory Activities.

^d^PRR: proportional reporting ratio.

^e^ROR: reporting odds ratio.

^f^IC: information component.

## Discussion

### Principal Findings

It is necessary to identify unknown AEs to reduce drug-related health risks [31]. Many efforts have been made to systematically detect unknown AEs using both social media platforms and traditional pharmacovigilance systems [32,33]. The rich amount of publicly available health information that patients post online is an invaluable additional data source for postmarket safety surveillance [24,32]. Therefore, researchers have used data from both social media and pharmacovigilance systems to detect AEs, as each type of data source has its strengths and limitations [34-36].

FAERS is the national pharmacovigilance system used in the United States, while WebMD is among the social media platforms most frequently used in research to retrieve patient reviews of drug experiences. Thus, we used these 2 data sets to detect new tramadol-related AEs in this study. Our 2 data sets evidenced several AEs known to be symptomatic of serotonin syndrome, including altered mental status (eg, agitation, anxiety, hallucinations), autonomic instability (eg, arrhythmias, tachycardia, labile blood pressure, hyperthermia), neuromuscular abnormalities (eg, hyperreflexia, tremor, rigidity), and gastrointestinal symptoms (eg, nausea, vomiting, diarrhea) [37,38]. However, there were qualitative differences in the reported tramadol-related AEs between the 2 data sets.

The FAERS data set produced a more diverse range of AEs than WebMD, in terms of both the range of severity and the affected organ systems. For example, reports of overdose, death, and completed suicide were only found on FAERS. FAERS reports also included 4 more SOCs in which tramadol-related AEs were reported than WebMD. Further, all but 1 (pain) of the new tramadol-related AEs detected was only found in the FAERS data set. Patients’ reviews on WebMD were primarily concerned with mild AEs such as pruritus, constipation, and hyperhidrosis. These differences can be explained by the difference in sample sizes and AE reporters on the 2 sites. FAERS data had 10 times more reports of tramadol-related AEs than WebMD, allowing the inclusion of a wider range of AEs. This demonstrates the value of larger data sets when selecting social media platforms for drug-induced AE detection. Because AE reports on social media are only self-reported, serious AEs such as suicide and death cannot be reported on such platforms. However, because the risk perceptions associated with a given drug can differ between patients (mainly for WebMD) and health care professionals (mainly for FAERS), combining data from both populations may contribute to improved drug safety assessments.

The tramadol-related pain that was frequently reported in both data sets may be a previously unknown tramadol-related AE. The FDA drug label does not list pain among the potential tramadol-induced side effects. However, the statistical measures of disproportionality were over the threshold criteria of signal detection (PRR=2.2; ROR=2.2; IC=1.1). In addition, the patients’ descriptions indicated increases in pain after taking tramadol. This is to be distinguished from both the persistence of existing pain due to an ineffective analgesic effect and pain caused by withdrawal following drug discontinuation. Increased pain might indicate opioid-induced hyperalgesia, which is increased sensitivity to pain caused by exposure to opioids [39]. There have been several reported cases of tramadol-induced hyperalgesia in previous studies [40,41]. Our result suggests that clinical assessments that consider opioid-induced hyperalgesia may be of value when patients complain of increased pain following tramadol treatment [42].

We detected 20 new serious AEs possibly related to tramadol. A clear distinction between the different types of serious AEs was difficult due to the diversity of AEs and related organ systems; however, most seemed to be broadly symptomatic of vascular diseases (eg, splenic artery aneurysm, femoral artery aneurysm, iliac artery occlusion) and their complications (eg, cerebellar infarction, quadriplegia, hemiplegia, paraplegia, paralysis). Our analysis also detected various coronary-related AEs. These results are not presented here because myocardial ischemia is already listed on the FDA drug label. The biological mechanisms that might explain an association between tramadol and vascular disease are not clear. A possible explanation is that tramadol mediates vascular homeostasis and thrombosis formation by inhibiting the reuptake of serotonin, affecting the platelet aggregation process [43]. Previous in vivo and in vitro studies have shown that tramadol use may enhance plasma coagulation and inhibit platelet disaggregation [44,45]. This finding from our analysis suggests that additional caution may be indicated before the use of tramadol in patients with vascular diseases as well as coronary artery disease.

### Limitations

Our study had some limitations. First, our data may have produced nonrepresentative figures describing the occurrence of AEs due to limitations of spontaneous reporting systems such as under-reporting, selective reporting, biases in patient’s drug preferences, and heterogeneity of the reports of different reporters [46]. Second, the results of our disproportionality analysis cannot prove a causal relationship. This method used the occurrence of AEs related to other drugs in the data as a proxy for the background incidence of AEs [47]. Thus, it may have been influenced by absolute report numbers. Third, posts from WebMD may have been misclassified into the wrong PTs due to a lack of clinical information. For example, if a patient complained that a coma occurred after taking tramadol, it cannot be medically confirmed that tramadol caused the coma without clinical information. Fourth, personal information from our data sets is based on voluntary reporting, and information has not been verified. Therefore, we could not rule out misinformation on demographics. Fifth, the user coverage may differ between 2 data sets, especially for the country profiles.

### Conclusions

Despite the several study limitations, our study has 2 main strengths. First, this study found new tramadol-related signals. Based on the essential feature of pharmacovigilance signal [14], these findings have political implications for preventing drug-related health harms through the early detection of adverse drug reactions. Second, this study found the additional possible AE, pain, by comparing data from social media and conventional pharmacovigilance. Because pain is the main indication for analgesics, including tramadol, it is difficult to classify it as an AE without considering the patient’s detailed symptom descriptions. Although FAERS is representative data for signal detection, detailed descriptions of AEs are not collected in this system. Thus, this study can be an example of the usefulness of patient drug reviews in social media in detecting unexpected AEs. However, the small sample size from WebMD prevented the detection of rare AEs by this means. To expand the utility of rare AE detection using social media, it would be necessary to use large amounts of social media data from several different sites, such as Twitter and Reddit. Social media data analysis using automatic natural language processing is a subject worthy of further research.

## Appendix

Multimedia Appendix 1 The number of tramadol-related adverse events in the US Food and Drug Administration (FDA) Adverse Event Reporting System (FAERS) data set.

Multimedia Appendix 2 Signal detections of adverse events related to tramadol in the US Food and Drug Administration (FDA) Adverse Event Reporting System (FAERS) data set.

## Abbreviations

AE: adverse event
FAERS: US Food and Drug Administration (FDA) Adverse Event Reporting System
IC: information component
MedDRA: Medical Dictionary for Regulatory Activities
PRR: proportional reporting ratio
PT: preferred term of the Medical Dictionary for Regulatory Activities
ROR: reporting odds ratio
SOC: system organ classes of the medical dictionary for regulatory activities
