# Supplementary material for: Comparison of Online Patient Reviews and National Pharmacovigilance Data for Tramadol-Related Adverse Events: Comparative Observational Study
Source: JMIR Public Health Surveill. 2022 Jan 4;8(1):e33311. doi: 10.2196/33311 (PMC8767477; doi:10.2196/33311)
Supplement: Multimedia Appendix 1 [file publichealth_v8i1e33311_app1.docx]

|  | **N** | **2016** | **2017** | **2018** | **2019** | **2020** |
| --- | --- | --- | --- | --- | --- | --- |
| **Total AE reports** | 7,843,727 | 1,304,836 | 1,369,522 | 1,684,751 | 1,727,249 | 1,757,369 |
| **N of identifying case** | 6,874,999 | 1,146,534 | 1,209,835 | 1,428,062 | 1,461,014 | 1,629,554 |
| **Tramadol AEs** | 92,127 | 14,662 | 13,922 | 16,682 | 20,598 | 26,263 |
| **Primary & Secondary suspected drug** ^a^ | 29,345 | 2,797 | 3,273 | 4,982 | 6,405 | 11,888 |
| Contramal | 326 | 45 | 47 | 51 | 83 | 100 |
| Conzip | 16 | . | . | 4 | 10 | 2 |
| Durela | 4 | 1 | . | 2 | . | 1 |
| Ralivia | 16 | 1 | . | . | 4 | 11 |
| Ryzolt | 645 | . | . | . | . | 645 |
| Tiparol | 8 | 2 | 3 | 1 | 2 | . |
| Tradolan | 27 | 4 | 2 | 14 | 5 | 2 |
| Tradonal Odis | 78 |  | 4 | 21 | 24 | 29 |
| Tramal | 509 | 107 | 115 | 138 | 74 | 75 |
| Tridural | 10 | . | . | 4 | 4 | 2 |
| Ultracet | 158 | 27 | 32 | 34 | 30 | 35 |
| Ultram | 1,757 | 243 | 249 | 379 | 371 | 515 |
| Zydol | 3 | 1 | 2 | . | . | . |
| Zytram | 6 | 1 | . | . | . | 5 |
| Tramadol ^b^ | 26,234 | 2,397 | 2,849 | 4,364 | 5,846 | 10,778 |

^a^ This number is smaller than the total sum of each medicine report because some reports were related to multiple medicine.

^b^ Including Tramadolum
