# Supplementary material for: Comparison of Online Patient Reviews and National Pharmacovigilance Data for Tramadol-Related Adverse Events: Comparative Observational Study
Source: JMIR Public Health Surveill. 2022 Jan 4;8(1):e33311. doi: 10.2196/33311 (PMC8767477; doi:10.2196/33311)
Supplement: Multimedia Appendix 2 [file publichealth_v8i1e33311_app2.docx]

| **SOC** | **PT** | **PRR** | **ROR** | **IC** |
| --- | --- | --- | --- | --- |
| Blood and lymphatic system disorders | Thymus disorder | 53.2 | 53.2 | 3.6 |
| Blood and lymphatic system disorders | Bandaemia | 21.5 | 21.5 | 2.9 |
| Blood and lymphatic system disorders | Splenic cyst | 13.7 | 13.7 | 2.3 |
| Blood and lymphatic system disorders | Hyperleukocytosis | 3.8 | 3.8 | 1.7 |
| Blood and lymphatic system disorders | Eosinophilia | 3.2 | 3.2 | 1.6 |
| Cardiac disorders | Toxic cardiomyopathy | 22.9 | 22.9 | 3.0 |
| Cardiac disorders | Cardiorenal syndrome | 10.0 | 10.0 | 2.8 |
| Cardiac disorders | Kounis syndrome | 5.4 | 5.4 | 2.2 |
| Cardiac disorders | Cardiomegaly | 3.0 | 3.0 | 1.5 |
| Ear and labyrinth disorders | Hyperacusis | 13.2 | 13.2 | 3.5 |
| Ear and labyrinth disorders | Ear haemorrhage | 4.7 | 4.7 | 2.1 |
| Endocrine disorders | Empty sella syndrome | 34.5 | 34.5 | 3.5 |
| Endocrine disorders | Lymphocytic hypophysitis | 6.6 | 6.6 | 2.0 |
| Eye disorders | Binocular eye movement disorder | 9.8 | 9.8 | 2.1 |
| Eye disorders | Cystoid macular oedema | 8.6 | 8.6 | 2.9 |
| Eye disorders | Accommodation disorder | 7.7 | 7.7 | 2.3 |
| Eye disorders | Gaze palsy | 3.4 | 3.4 | 1.5 |
| Eye disorders | Angle closure glaucoma | 2.7 | 2.7 | 1.3 |
| Eye disorders | Macular oedema | 2.6 | 2.6 | 1.3 |
| Eye disorders | Photophobia | 2.0 | 2.0 | 1.0 |
| Gastrointestinal disorders | Splenic artery aneurysm | 15.8 | 15.8 | 2.5 |
| Gastrointestinal disorders | Dumping syndrome | 10.7 | 10.7 | 2.3 |
| Gastrointestinal disorders | Burning mouth syndrome | 10.6 | 10.6 | 2.8 |
| Gastrointestinal disorders | Gastric mucosal lesion | 8.4 | 8.4 | 2.5 |
| Gastrointestinal disorders | Lip discolouration | 8.3 | 8.3 | 2.7 |
| Gastrointestinal disorders | Pancreatic cyst | 4.3 | 4.3 | 1.9 |
| Gastrointestinal disorders | Volvulus | 4.0 | 4.0 | 1.8 |
| Gastrointestinal disorders | Large intestine perforation | 2.5 | 2.5 | 1.2 |
| General disorders and administration site conditions | Precancerous condition | 19.4 | 19.4 | 3.1 |
| General disorders and administration site conditions | Breakthrough pain | 5.0 | 5.0 | 2.1 |
| General disorders and administration site conditions | Facial pain | 2.6 | 2.6 | 1.3 |
| General disorders and administration site conditions | Pain | 2.2 | 2.2 | 1.1 |
| Immune system disorders | Serum sickness | 3.4 | 3.4 | 1.6 |
| Infections and infestations | Herpes zoster oticus | 25.8 | 25.9 | 3.8 |
| Infections and infestations | Cardiac infection | 5.5 | 5.5 | 2.0 |
| Infections and infestations | Empyema | 4.6 | 4.6 | 2.0 |
| Infections and infestations | Endocarditis | 3.5 | 3.5 | 1.7 |
| Investigations | Pain threshold decreased | 37.7 | 37.7 | 3.2 |
| Investigations | Prothrombin level increased | 20.1 | 20.1 | 3.1 |
| Investigations | Urine sodium increased | 14.8 | 14.8 | 2.3 |
| Investigations | Prothrombin time shortened | 8.0 | 8.0 | 2.7 |
| Investigations | Blood osmolarity decreased | 7.5 | 7.5 | 2.2 |
| Investigations | Alanine aminotransferase abnormal | 5.0 | 5.0 | 2.1 |
| Investigations | Prothrombin level decreased | 4.9 | 4.9 | 1.8 |
| Investigations | Anion gap increased | 4.7 | 4.7 | 2.0 |
| Investigations | Blood immunoglobulin e increased | 4.6 | 4.6 | 2.0 |
| Investigations | Urine output increased | 4.5 | 4.6 | 2.0 |
| Investigations | Red blood cell count increased | 3.1 | 3.1 | 1.5 |
| Investigations | Bone density abnormal | 2.4 | 2.4 | 1.2 |
| Metabolism and nutrition disorders | Acid-base balance disorder mixed | 46.5 | 46.5 | 2.9 |
| Metabolism and nutrition disorders | Hyperlipasaemia | 17.4 | 17.5 | 3.4 |
| Metabolism and nutrition disorders | Hyperammonaemia | 6.4 | 6.4 | 2.6 |
| Metabolism and nutrition disorders | Cell death | 4.8 | 4.8 | 2.1 |
| Metabolism and nutrition disorders | Metabolic acidosis | 4.2 | 4.2 | 2.0 |
| Metabolism and nutrition disorders | Hyperlactacidaemia | 3.2 | 3.2 | 1.6 |
| Metabolism and nutrition disorders | Hypercholesterolaemia | 2.5 | 2.5 | 1.3 |
| Metabolism and nutrition disorders | Hypokalaemia | 2.1 | 2.1 | 1.1 |
| Musculoskeletal and connective tissue disorders | Ligament calcification | 348.9 | 349.0 | 4.1 |
| Musculoskeletal and connective tissue disorders | Sacroiliitis | 41.0 | 41.2 | 4.9 |
| Musculoskeletal and connective tissue disorders | Metatarsalgia | 15.1 | 15.1 | 2.7 |
| Musculoskeletal and connective tissue disorders | Bursa disorder | 14.4 | 14.4 | 2.8 |
| Musculoskeletal and connective tissue disorders | Myofascial pain syndrome | 11.1 | 11.1 | 2.8 |
| Musculoskeletal and connective tissue disorders | Haematoma muscle | 9.0 | 9.0 | 2.8 |
| Musculoskeletal and connective tissue disorders | Periarthritis | 6.0 | 6.0 | 2.4 |
| Musculoskeletal and connective tissue disorders | Facet joint syndrome | 5.8 | 5.8 | 1.9 |
| Musculoskeletal and connective tissue disorders | Neuropathic arthropathy | 5.7 | 5.7 | 2.1 |
| Musculoskeletal and connective tissue disorders | Fibromyalgia | 5.5 | 5.5 | 2.4 |
| Musculoskeletal and connective tissue disorders | Sjogren's syndrome | 4.1 | 4.1 | 1.9 |
| Musculoskeletal and connective tissue disorders | Rhabdomyolysis | 3.7 | 3.7 | 1.9 |
| Musculoskeletal and connective tissue disorders | Rotator cuff syndrome | 2.5 | 2.5 | 1.3 |
| Musculoskeletal and connective tissue disorders | Pathological fracture | 2.2 | 2.2 | 1.1 |
| Nervous system disorders | Neurovascular conflict | 139.6 | 139.6 | 2.7 |
| Nervous system disorders | Acute motor-sensory axonal neuropathy | 37.8 | 37.8 | 4.2 |
| Nervous system disorders | Central pain syndrome | 20.3 | 20.3 | 3.3 |
| Nervous system disorders | Cholinergic syndrome | 18.3 | 18.3 | 3.5 |
| Nervous system disorders | Anticholinergic syndrome | 14.4 | 14.4 | 3.4 |
| Nervous system disorders | Meralgia paraesthetica | 13.7 | 13.7 | 2.3 |
| Nervous system disorders | Lumbar radiculopathy | 10.5 | 10.5 | 2.9 |
| Nervous system disorders | Hand-eye coordination impaired | 9.6 | 9.6 | 2.1 |
| Nervous system disorders | Allodynia | 9.5 | 9.5 | 2.7 |
| Nervous system disorders | Cervical radiculopathy | 9.5 | 9.5 | 2.7 |
| Nervous system disorders | Areflexia | 7.5 | 7.5 | 2.7 |
| Nervous system disorders | Metabolic encephalopathy | 5.8 | 5.8 | 2.4 |
| Nervous system disorders | Pleocytosis | 5.7 | 5.7 | 2.0 |
| Nervous system disorders | Cerebellar infarction | 4.9 | 4.9 | 2.0 |
| Nervous system disorders | Cerebral vasoconstriction | 4.8 | 4.8 | 1.8 |
| Nervous system disorders | Quadriplegia | 4.5 | 4.5 | 2.0 |
| Nervous system disorders | Hyperaesthesia | 4.2 | 4.2 | 2.0 |
| Nervous system disorders | Neuralgia | 3.7 | 3.7 | 1.9 |
| Nervous system disorders | Radiculopathy | 3.6 | 3.6 | 1.7 |
| Nervous system disorders | Complex regional pain syndrome | 3.3 | 3.3 | 1.5 |
| Nervous system disorders | Toxic encephalopathy | 3.2 | 3.2 | 1.6 |
| Nervous system disorders | Facial paresis | 2.9 | 2.9 | 1.4 |
| Nervous system disorders | Leukoencephalopathy | 2.9 | 2.9 | 1.4 |
| Nervous system disorders | Hemiplegia | 2.7 | 2.7 | 1.4 |
| Nervous system disorders | Cerebral haematoma | 2.7 | 2.7 | 1.3 |
| Nervous system disorders | Trigeminal neuralgia | 2.5 | 2.5 | 1.2 |
| Nervous system disorders | Parosmia | 2.5 | 2.5 | 1.3 |
| Nervous system disorders | Restless legs syndrome | 2.4 | 2.4 | 1.2 |
| Nervous system disorders | Paraplegia | 2.3 | 2.3 | 1.1 |
| Nervous system disorders | Monoplegia | 2.1 | 2.1 | 1.0 |
| Nervous system disorders | Hypotonia | 2.1 | 2.1 | 1.0 |
| Nervous system disorders | Drooling | 2.1 | 2.1 | 1.0 |
| Nervous system disorders | Paralysis | 2.1 | 2.1 | 1.0 |
| Renal and urinary disorders | Bladder hypertrophy | 8.4 | 8.4 | 2.3 |
| Renal and urinary disorders | Nephrosclerosis | 5.8 | 5.8 | 2.2 |
| Reproductive system and breast disorders | Perineal fistula | 67.8 | 67.9 | 3.6 |
| Reproductive system and breast disorders | Genital erosion | 31.7 | 31.7 | 3.5 |
| Reproductive system and breast disorders | Benign prostatic hyperplasia | 3.9 | 3.9 | 1.9 |
| Respiratory, thoracic and mediastinal disorders | Alveolar lung disease | 65.5 | 65.6 | 4.5 |
| Respiratory, thoracic and mediastinal disorders | Diffuse alveolar damage | 15.0 | 15.0 | 3.5 |
| Respiratory, thoracic and mediastinal disorders | Pneumothorax spontaneous | 7.4 | 7.5 | 2.4 |
| Respiratory, thoracic and mediastinal disorders | Hiccups | 2.8 | 2.8 | 1.5 |
| Skin and subcutaneous tissue disorders | Haematidrosis | 232.6 | 232.6 | 3.1 |
| Skin and subcutaneous tissue disorders | Acantholysis | 40.5 | 40.5 | 2.9 |
| Skin and subcutaneous tissue disorders | Livedo reticularis | 8.4 | 8.4 | 2.8 |
| Skin and subcutaneous tissue disorders | Acute generalised exanthematous pustulosis | 3.1 | 3.1 | 1.6 |
| Skin and subcutaneous tissue disorders | Drug reaction with eosinophilia and systemic symptoms | 2.0 | 2.0 | 1.0 |
| Vascular disorders | Femoral artery aneurysm | 74.8 | 74.8 | 3.9 |
| Vascular disorders | Iliac artery occlusion | 34.5 | 34.5 | 3.8 |
| Vascular disorders | Arteriovenous fistula | 13.8 | 13.8 | 3.1 |
| Vascular disorders | Circulatory collapse | 3.9 | 3.9 | 1.9 |
| Vascular disorders | Angiopathy | 2.8 | 2.8 | 1.4 |
| Vascular disorders | Vasculitis | 2.5 | 2.5 | 1.3 |

SOC = system organ classes of the Medical Dictionary for Regulatory Activities

PT = preferred term of the Medical Dictionary for Regulatory Activities

PRR = proportional reporting ratio

ROR = reporting odds ratio

IC = information component.
